# Respiratory maze: An anatomical variant of tracheal bronchus

**DOI:** 10.1002/rcr2.1355

**Published:** 2024-04-24

**Authors:** Dedeepya Gullapalli, Shivendra Tangutoori, Subramanya Shyam Ganti

**Affiliations:** ^1^ Internal Medicine Appalachian Regional Healthcare Internal Medicine Residency Program Harlan Kentucky USA; ^2^ Department of Pulmonary Critical Care Medicine Appalachian Regional Healthcare Harlan Kentucky USA

**Keywords:** abnormal CT chest, anatomical variant, bronchu suis, flexible bronchoscopy, tracheobronchus

## Abstract

This case highlights an uncommon anatomical variation in the airway known as Tracheal bronchus, which can sometimes lead to recurrent pneumonia. It is crucial to exercise caution during intubation in patients with this condition.

## Graphical abstract

Tracheal bronchus is a rare congenital abnormality of bronchial variation arising from trachea. Special attention should be paid during intubation as it can potentially obstruct the bronchus.

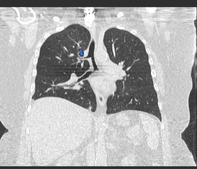

## CLINICAL IMAGE

**FIGURE 1:**
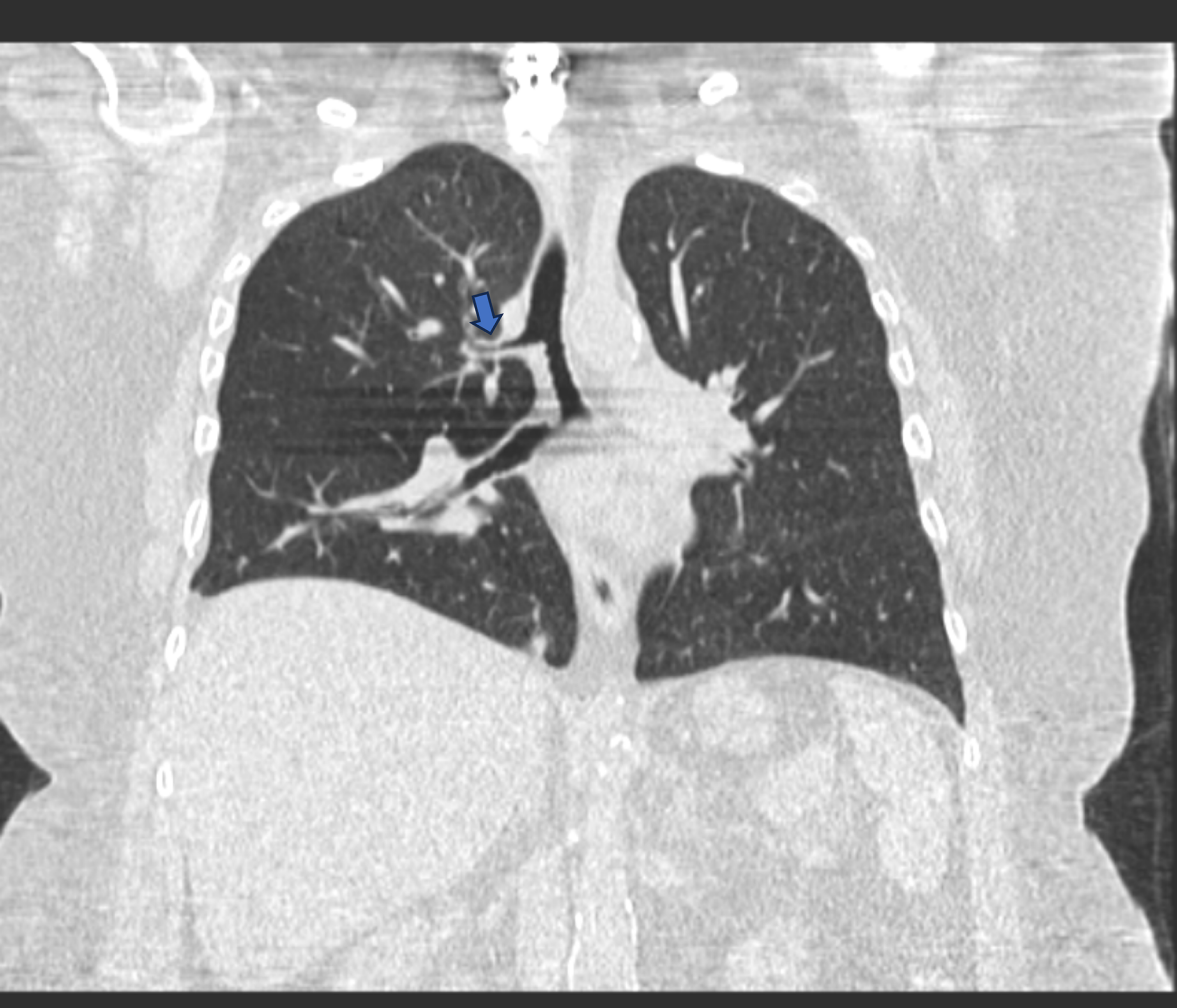
Computed tomography chest, arrow mark indicating the right tracheal bronchus.

A 59‐year‐old female patient presented to the pulmonology clinic for evaluation of chronic shortness of breath for the past 1 year. Associated with cough and whitish‐coloured phlegm. She has 2 pillow orthopnea, paroxysmal dyspnea, leg swelling, and occasional wheezes present. No aggravating or relieving factors were noted. The patient is smoking 1 pack per day, 52 packyears, failed to quit smoking. Pulmonary Function Test based on GOLD criteria revealed moderately obstructive lung disease with air trapping. The patient was started on a tiotropium‐olodaterol inhaler and as needed albuterol inhaler. Lung cancer screening with low‐dose CT showed an incidentally noted tracheal bronchus supplying the right upper lobe, representing a normal anatomic variant (Figure 1). The patient was never admitted with pneumonia. We educated the patient on requiring special attention if the patient needs to be intubated considering her anatomical airway variant. Flexible bronchoscopy should be performed to find the location of the tracheal bronchus. Either double‐lumen tube or Univent tube with bronchial blocker or regular tracheal tube with bronchial blocker are effective in providing successful lung isolation as it allows the air through main airway(trachea) rather than through tracheal bronchus. Asymptomatic cases require observation. Segmentectomy and lobectomy are performed for the treatment of recurrent pneumonia. Because of the multimodal treatment approach, tracheal bronchus has a good prognosis.^1^

## AUTHOR CONTRIBUTIONS

Dedeepya Gullapalli contributed to the structure forming and data collection, Shivendra Tangutoori contributed for the collection of image and consent from the patient, Subramanya Shyam Ganti contributed to the amendments in the image.

## CONFLICT OF INTEREST STATEMENT

None declared.

## ETHICS STATEMENT

Our institution does not require ethical approval for reporting individual cases or case series. The authors declared that appropriate written informed consent was obtained for the publication of this manuscript and accompanying images.

## Data Availability

N/A.
